# Furin Functions as a Nonproteolytic Chaperone for Matrix Metalloproteinase-28: MMP-28 Propeptide Sequence Requirement

**DOI:** 10.1155/2011/630319

**Published:** 2010-11-01

**Authors:** Maria Pavlaki, Stanley Zucker, Antoine Dufour, Nikki Calabrese, Wadie Bahou, Jian Cao

**Affiliations:** ^1^Divsions of Cancer Prevention, Department of Medicine, Stony Brook University, Stony Brook, NY 11794, USA; ^2^Division Hematology and Oncology, Department of Medicine, Stony Brook University, Stony Brook, NY 11794, USA; ^3^Department of Research, Veterans Affairs Medical Center, Northport, NY 11768, USA; ^4^Department of Chemistry, Stony Brook University, Stony Brook, NY 11794, USA

## Abstract

Although MMP-28 is involved in numerous important physiologic and pathologic conditions, the mechanisms of action of this secreted proteinase is not well understood. We now have demonstrated that furin serves as an intermolecular chaperone for MMP-28 secretion by interacting with the propeptide domain of MMP-28. Employing COS-1 cells transfected with MMP-28 cDNA, protein levels of MMP-28 were quite low in conditioned media as compared to cell lysates. Coexpression of MMP-28 with furin cDNA resulted in markedly enhanced MMP-28 secretion. Contrary to expectation, cleavage of MMP-28 at the furin consensus sequence did not occur and proteolytic inactive furin was equally effective in enhancing MMP-28 secretion. Furin and MMP-28 coimmunoprecipitated and were partially coimmunolocalized in the cytoplasm of transfected cells. Cotransfection with furin cDNA also enhanced MMP-28 induced cell migration. In conclusion, our data provide a novel mechanism for MMP-28 function in cells in which furin serves as an intermolecular chaperone.

## 1. Introduction

In the four decades following the discovery of an amphibian collagenase [1], our understanding of matrix metalloproteinases (MMPs) has evolved from distinction between MMP family members based on substrate preferences, to nucleotide and amino acid sequencing, and most recently, to structural-functional characteristics [2, 3]. MMPs are implicated in extracellular matrix degradation, cell migration, proliferation, apoptosis, and tissue remodeling in numerous biologic and pathologic conditions [2–5]. The basic structure of the zinc-binding region within the catalytic domain and the cysteine switch sequence within the propeptide domain is preserved in 23 of 24 human MMPs [5]. All six membrane type MMPs (MT-MMPs) also contain a paired basic amino acid sequence (Arg*-*X*-*Lys*/*Arg*-*Arg) located between the N-terminal propeptide domain and catalytic domain. This unique motif facilitates cleavage and activation of these proteinases by proprotein convertases, such as furin [6]. The furin activation sequence has also been identified in selected secreted MMPs including MMP-11, MMP-21, MMP-23, and the most recently identified, MMP-28 [7–9]. 

Furin, a ubiquitously expressed member of the proprotein/convertase family, primarily functions in cells by processing inactive precursor proteins to their functional/mature forms [10, 11]. Less often, furin processing of propeptides (proADAMTS9 and proMMP-2) can diminish function of the mature protein [12, 13]. Furin is localized primarily in the *trans*-Golgi network (TGN), a late Golgi structure that is responsible for sorting secretory pathway proteins to their final destinations. From the TGN, furin follows a highly regulated trafficking itinerary through several TGN/endosomal compartments and the cell surface [14]. Furin cleaves proproteins on the C-terminal side of the consensus sequence -Arg-*X*-Lys/Arg-Arg in the TGN [15]. Arg residues at the P1 and P4 positions of the cleavage site are essential, whereas the P2 basic amino acid is not essential, but serves to enhance processing efficiency. Hence, R*XX*R represents the minimal furin cleavage site. Furin is upregulated in several cancers, including nonsmall cell lung carcinomas, squamous cell carcinomas of the head and neck, breast carcinomas, ovarian carcinomas, and glioblastomas [16–22]. Elevated expression of furin results in increased tumor cell migration, invasion, and metastasis. In addition to conversion of proproteins to activated forms, furin has also been found to promote protein trafficking, including latent MT1-MMP [23]; the mechanism underlying enhanced proprotein trafficking remains to be understood. 

Human MMP-28 [24] was initially designated as epilysin based on presumed specific expression in keratinocytes. Furin was subsequently cloned from human testis [24] and lung cDNA libraries [25]. The protein contains all of the typical MMP domains. MMP-28 is also expressed to a lesser degree in other normal, intact tissues including the gastrointestinal tract, lung, kidney, heart, skeletal muscle, and nervous system. Recombinant MMP-28 has been reported to degrade casein [24], Nogo-A (a myelin component), NCAM-1 [26] and slowly cleaves a pan-MMP fluorogenic substrate [26]. With the exception of keratinocytes, basal expression of MMP-28 has been reported to be quite low among other cell lines [27]. Among 12 growth factors tested, only tumor necrosis factor *α* has been reported to upregulate MMP-28 mRNA; MMP-28 expression appears not to be modulated by extracellular matrices. MMP-28 expression is tightly regulated and has been implicated in tissue homeostasis [24, 28], in cell proliferation during wound repair [27], and in neuronal development and demyelination [26]. Little is known about in vivo substrates and activators of MMP-28 [27]. Xenopus MMP-28 has been demonstrated to be activated by furin in vivo [26]. Illman et al. [29] reported that overexpression of MMP-28 in lung adenocarcinoma cells induced an epithelial-to-mesenchymal transition and cell invasion requiring the catalytic activity of MMP-28 and cell surface localization mediated by the hemopexin domain of MMP-28. This epithelial-to-mesenchymal transition involved activation of the TGF-*β* mechanism followed by upregulation of MT1-MMP and MMP-9. Cell migration of these EMT cells was independent of MMP activity.

Although propeptide domains of MMPs are best known for maintaining the protease in an inactive state, we previously demonstrated that the propeptide domain of MT1-MMP functions as a intramolecular chaperone required for trafficking and function of the protease on the cell surface [30]. Alanine substitution mutations revealed that a conserved tetrapeptide (Tyr^42^-Gly^43^-Tyr^44^-Leu^45^) within the propeptide of MT1-MMP was crucial for the chaperone function of the prodomain involving trafficking of functional MT1-MMP to the plasma membrane [31]. Of interest, the YGYL tetrapeptide is fully conserved in human as well as rat, mouse, and Xenopus laevis MMP-28 [9, 26]. The function of the YGYL in MMP-28 is still unknown. Based on the unique function of this peptide in MT1-MMP [31], we have herein examined the role of YGYL in the propeptide domain of MMP-28. An important function for the YL motif on cell secretion of MMP-28 has been demonstrated. The formation of an intracellular protein complex between latent furin and the prodomain of MMP-28 appears to play a critical role in secretion and function of this protease. 

## 2. Materials and Methods

### 2.1. Reagents

Oligo primers were purchased from Operon (Al, Huntsville). pcDNA3.1 and expression vectors were purchased from Invitrogen (Carlsbad, CA). Anti-Myc monoclonal antibody was purchased from Roche (Indianapolis, IN). Antifurin polyclonal antibodies were purchased from Santa Cruz (Santa Cruz, CA). Anti-MT1-MMP hinge antibody was purchased from Triple Point Biologics (Forest Grove, OR). Anti-tubulin antibody was purchased from Cell Signaling Technology (Davers, MA). Alexa 568-conjugated goat anti-mouse IgG was purchased from Invitrogen (Carlsbad, CA). Endoglycosidase H and peptide:N-glycosidase F were purchased form Roche (Indianapolis, IN) and NEB (Ipswich, MA), respectively. Wild-type, soluble, and dominant negative furin (furin^S-A^) cDNAs were described previously [32]. MT1-MMP, its chimeras [33] and MMP-28 cDNA [24] were previously reported.

### 2.2. Cell Culture and Transfection

COS-1 cells were purchased from ATCC (Manassas, VA) and were maintained in Dulbecco's modified Eagle's medium (Invitrogen). Plasmids were transfected into COS-1 cells using Transfectin reagent (Bio-Rad, CA). Transfected cells were incubated for 48 h at 37°C in DMEM containing 10% fetal calf serum (FCS).

### 2.3. Construction of Plasmids

MMP-28 with a carboxy-terminal Myc tag (MMP-28/Myc) was generated using the pcDNA3.1 expression vector (Invitrogen). The MMP-28 cDNA containing the open reading frame of MMP-28 was amplified by a PCR approach using the primers sets: forward primer, number 1086: 5′-3′: AAGAATTCCGGCGAGATGGTCGCGCGCGTC and reverse primer, number 1117: 5′-3′ AAAAGCTTGAACAGGGCGCTCCCCGAGTTGGC. The resultant PCR fragment was then cloned into the pcDNA3.1 vector at EcoR I and Hind III sites to generate MMP-28/Myc chimeric cDNA. 

To construct a constitutively inactive MMP-28/Myc (MMP-28^E241A^/Myc), the glutamic acid (E^241^) of MMP-28 was converted to alanine by a PCR-based mutagenesis approach using wild-type MMP-28/Myc as a template with mutagenesis primers (forward primer, no. 1166, 5′-3′: TGGTGCTGGCGCACGCGATCGGTCACACGC and reverse primer, no. 1167, 5′-3′: GCGTGTGACCGATCGCGTGCGCCAGCACC) (underline letters represent mutation site) (Quick Change Site-Directed Mutagenesis kit, Stratagene) to generate MMP-28^E241A^. Using a similar approach, MMP28^R119KAA^, MMP28^Y43G44-AA^, and MMP28^Y45L46-AA^ were generated with primer sets as follow: MMP28^R119KAA^ (forward, 5′-3′: ACCAAACTGAGGCGTAAGGCAGCCTTTGCAAAGCAAGGTAA, and reverse primer, 5′-3′: TTACCTTGCTTTGCAAAGGCTGCCTTACGCCTCATTTTGGT); MMP28^Y43G44-AA^ (forward primer, 5′-3′: GCGGAGGCATTCCT AGAGAAGGCCGCATACCTCAATGAACAGGTCCCC, and reverse primer, 5′-3′: GGGGACCT GTTCATTGAGGTATGCGGCCTTCTCTAGGAATGCCTC CGC); and MMP28^Y45L46-AA^ (forward primer, 5′-3′: GCATTCCTAGAGAAGTACGGAGCCGCCAATGAACAGGTCCCCAAAGCT; and reverse primer, 5′-3′: AGCTTTGGGGACCTG TTCATTGGCGGCTCCGTACTTCTC TAGGAATGC). All constructs were verified by DNA sequencing.

### 2.4. Immunofluorescent Staining and Laser Scanning Confocal Microscopy

Cultured cells were fixed with 4% Paraformaldehyde (PFA)/PBS followed by blocking with 3% BSA/PBS. MMP28-Myc and furin were detected by an antibody against the Myc tag (MMP-28) and furin followed by secondary antibodies conjugated with Alexa 568 and FITC (Invitrogen). The immunostained cells were examined using a Zeiss LSM 510 META NLO Two-Photon Laser Scanning Confocal Microscope System.

### 2.5. Co-Immunoprecipitation

COS-1 cells cotransfected with furin along with MMP-28/Myc or MT1-MMP/Myc cDNAs were lysed with RIPA lysis buffer. An aliquot of the original sample was also saved to assess protein expression in the transfected cells by a Western blotting. The cell lysates were immunoprecipitated with antifurin antibodies followed by capturing antigen-antibody complex with protein A agarose beads (Invitrogen). Myc tagged MMP-28 and MT1-MMP complexes were fractionated by SDS-PAGE (10% polyacrylamide gel) and Western blotting was performed using anti-Myc antibodies. In reciprocal coimmunoprecipitation experiments, cell lysates were immunoprecipitated with anti-Myc antibodies followed by Western blotting with antifurin antibodies.

### 2.6. Digestion with EndoHand PNGase F

The cell lysate of COS-1 cells transfected with MMP-28 and furin was boiled for 10 min in denaturing buffer containing 1% 2-mercaptoethanol and 0.5% SDS to expose fully all glycosylation sites, and then deglycosylation was done by treatment with Endo H or PNGase F at 37°C for 3 h. The buffers used in these enzyme reactions were 50 mM sodium citrate (pH 5.5) for Endo H and 50 mM sodium phosphate (pH 7.5) containing 1% Nonidet P-40 for PNGase F. The samples were then separated by SDS-PAGE followed by Western blotting using anti-Myc antibody.

### 2.7. Transwell Migration Assay

Polycarbonate membranes of 13 mm diameter with 8 *μ*m pore size (Neuro Probe, MD) were inserted into the Blind-Well Chemotactic chambers (Neuro Probe, MD). Prior to seeding into the transwell inserts, COS-1 cells were released from plates with trypsin-EDTA and 2 × 10^4^ cells/well placed in the top chamber of transwell migration chambers in triplicates. The lower chamber was filled with DMEM containing 10% FCS (200 *μ*L). After 6 hours, COS-1 cells that had not migrated to the lower chamber were removed from the upper surface of the transwell membrane with a cotton swab. Migrating cells on the lower membrane surface were fixed, stained with 0.1% crystal violet, and examined under a microscope. The number of cells in 10 areas of the filters was counted to obtain the number of migrating cells. Each experiment was repeated three times.

### 2.8. Statistical Analysis

Data are presented as mean ± SEM. Employing GraphPad Prism 5, statistical significance was tested by using Student's *t*-test (with **P < .001 and ***P < .0005).

### 2.9. Procedures for Preparation of Cell Lysate and Western Blotting

Basic protocols for these techniques have been described in our recent paper [34]. 

## 3. Results

**Figure 1:**
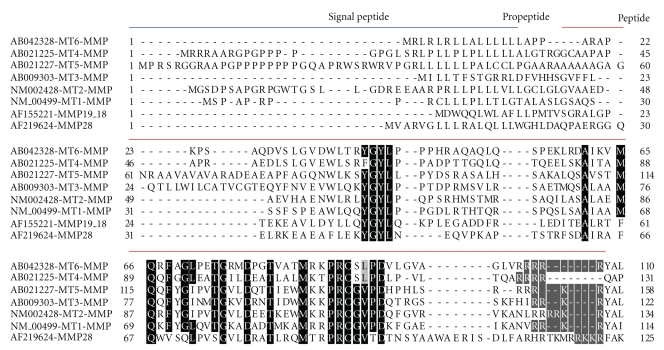
Alignment of the propeptide domain of YGYL containing MMPs. The intramolecular chaperone sequence of YGYL, cysteine switch sequence of PRCGVPD, and all conserved amino acids within the propeptide domains of MMPs are highlighted in black, and furin consensus sequence of RXXR motif is highlight in gray. Blue line represents a signal peptide and red line represents propeptide domain.

We previously demonstrated that the propeptide domain of MT1-MMP acts as an intramolecular chaperone required for trafficking and function of the proteinase on the plasma membrane [30, 35, 36]. We further demonstrated that a conserved ^42^YGYL^45^ sequence, which is present within the propeptide of all six members of the MT-MMP subfamily, appears critical for the intramolecular chaperone function [31]. By analyzing the amino acid sequence of secretory MMPs, the propeptide domains of MMP-19 and -28 were noted to contains the same YGYL sequence and a RXXR furin consensus motif as displayed in MT-MMPs (Figure 1). These observations have led us to ponder whether the YGYL sequence confers an analogous function in the secretion of MMP-28.

### 3.1. Expression of MMP-28 in COS-1 Cells: Furin Facilitates Secretion of MMP-28

**Figure 2:**
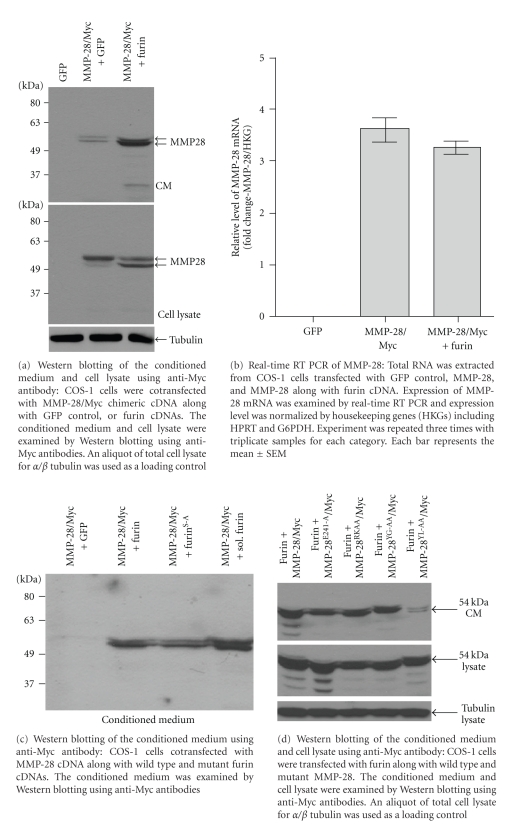
Enhanced secretion of MMP-28 by co-transfection of COS-1 cells with furin cDNA. The data show that MMP-28 secretion requires coexpression with furin. Furin does not increase newly synthesized MMP-28. Instead, Furin promotes existing MMP-28 secretion from storage pools to secretory pathway. Active state of furin is not prerequisite.

To determine the requirement of the YGYL motif within the propeptide domain in protein trafficking and secretion, MMP-28/Myc cDNA was transiently transfected into COS-1 cells followed by Western blotting using an anti-Myc antibody. Unexpectedly, (secreted) MMP-28/Myc was barely detected in the cell conditioned medium (Figure 2(a)); most of the recombinant protein was identified in the cell lysate. Two MMP-28 bands with approximate molecular mass of 56 kDa and 52 kDa were seen in the cell lysate (Figure 2(a)). Other experiments were performed to decipher the limited secretion of MMP-28. Diminished secretion of MMP-28 was not due to malfunction of the N-terminal signal peptide of MMP-28, since replacement of the signal peptide cDNA of MMP-28 by that of MT1-MMP did not enhance the secretion of MMP-28 chimera (data not shown). 

Since MMP-28 carries a RKKR furin consensus sequence between the propeptide and catalytic domains (Figure 1), we anticipated that furin in the *Trans* Golgi Network (TGN) would cleave and activate MMP-28 prior to secretion, resulting in two distinct molecules (see explanation below). To test this possibility, COS-1 cells were cotransfected with MMP-28/Myc along with furin or GFP (as control) cDNA. After 18 hours, the conditioned media and cell lysates were collected and immunoblotting was performed using an anti-Myc antibody. Unexpectedly, coexpression of MMP-28 with furin resulted in increased secretion of MMP-28 into conditioned media (Figure 2(a)). In contrast, the total amount of MMP-28 in the cell lysate did not appear to be affected by coexpression of furin (Figure 2(a)). Relative levels of MMP-28 mRNA were not affected by cotransfection with furin cDNA, which minimizes a role for furin in regulation of transcription (Figure 2(b)). 

Although the molecular weight difference between the doublet bands (56 and 52 kDa) of MMP-28 in the cell lysate was less than the ~10 kDa difference anticipated between latent and activated MMPs, additional studies were needed to determine if furin cleavage (activation) of proMMP-28 was required for enhanced secretion. To this end, we employed loss-of-function mutations of furin which were previously reported [12]. Transfection of COS-1 cells with inactive furin (Furin^S-A^) cDNA or soluble furin (Sol.Furin) cDNA [12] resulted in enhanced MMP-28 secretion; no effect on the apparent molecular weight of MMP-28 was noted between wild-type furin or mutant furin-transfected cells (Figure 2(c)). Furthermore, transfection of COS-1 cells with wild-type furin cDNA as well as MMP-28 cDNA containing an alanine disrupted furin consensus sequence (R^119^KAA instead of R^119^KKR) did not alter the secretion of the MMP-28 doublet protein bands (Figure 2(d)). Using another approach to determine if activation of proMMP-28 is required prior to secretion, a glutamic acid to alanine substitution mutation (E^241^-A) in the catalytic domain was employed as a technique to generate a nonproteolytic MMP [37]. Incubation of COS-1 cells transfected with MMP-28 E^241^-A cDNA or incubation of wild-type MMP-28 transfected COS-1 cells with a broad spectrum MMP inhibitor (BB94, data not shown) did not abrogate furin-induced secretion of MMP-28 (Figure 2(d)). Furthermore, the conditioned media from COS-1 cells transfected with MMP-28 or MMP-28 along with furin cDNAs did not show significant proteolytic activity using a fluorogenic substrate assay [38] (data not shown). These results suggest that furin promotes the secretion of MMP-28 in COS-1 cells without processing/cleavage of the latent form of the proteinase. 

**Figure 3:**
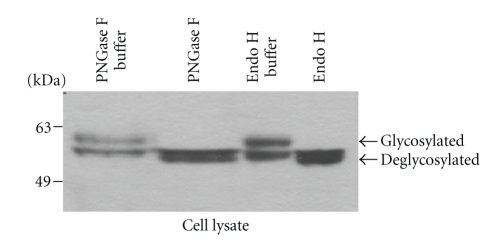
Deglycosylation of MMP-28 examined by Western blotting. The cell lysate of COS-1 cells transfected with MMP-28 along with furin cDNAs was treated with and without PNGase F or Endo H. The samples were then examined by Western blotting using anti-Myc antibodies.

In view of the lack of evidence for proteolytic cleavage to explain the doublet bands of MMP-28, we proceeded to explore the report that MMP-28 is N-glycosylated during secretory trafficking [39]. To test if MMP-28 expressed in COS-1 cells is glycosylated, the conditioned medium from COS-1 cells cotransfected with MMP-28 and furin cDNAs was treated with endoglycosidase H and peptide:N-glycosidase F (PNGase F). Endoglycosidase H cleaves asparagine-linked oligomannose and hybrid, but not complex, oligosaccharides from glycoproteins, generating a truncated sugar molecule with one N-acetylglucosamine residue remaining on the asparagine. In contrast, PNGase F cleaves asparagine-linked high mannose as well as hybrid and complex oligosaccharides from glycoproteins. In our experiments, both glycosidases resulted in conversion of the doublet of MMP-28 to the lower molecular weight band (Figure 3) suggesting that the higher band represents the N-glycosylated form of MMP-28. 

### 3.2. Requirement of the YL Motif for MMP-28 Secretion

To further delineate whether the YGYL amino acid sequence [31] in the propeptide domain of MMP-28 is required for secretion, a series of point mutations were generated using a site-directed mutagenesis approach. COS-1 cells were cotransfected with furin cDNA along with wild-type and mutant MMP-28/Myc cDNAs. Serum-free conditioned media were collected and MMP-28/Myc secretion was assessed. The tetrapeptide (YGYL) alanine substitution mutation resulted in decreased MMP-28 secretion (data not shown). Among the tetrapeptide of YGYL, the tyrosine-leucine (Y^45^L^46^) amino acid sequence in the propeptide of MMP-28 is critical for furin-induced secretion of MMP-28. In contrast to wild-type MMP-28, secreted MMP28^Y45L46-AA^ was not increased in the presence of cotransfection with furin cDNA 28 (Figure 2(d)). Mutation of the tyrosine-glycine (MMP28^Y43G44-AA^) amino acid sequence has no effect on MMP-28 secretion (Figure 2(d)). These results suggest that Tyr^45^-Leu^46^ motif is required for MMP-28 secretion.

### 3.3. Complex Formation between MMP-28 and Furin

**Figure 4:**
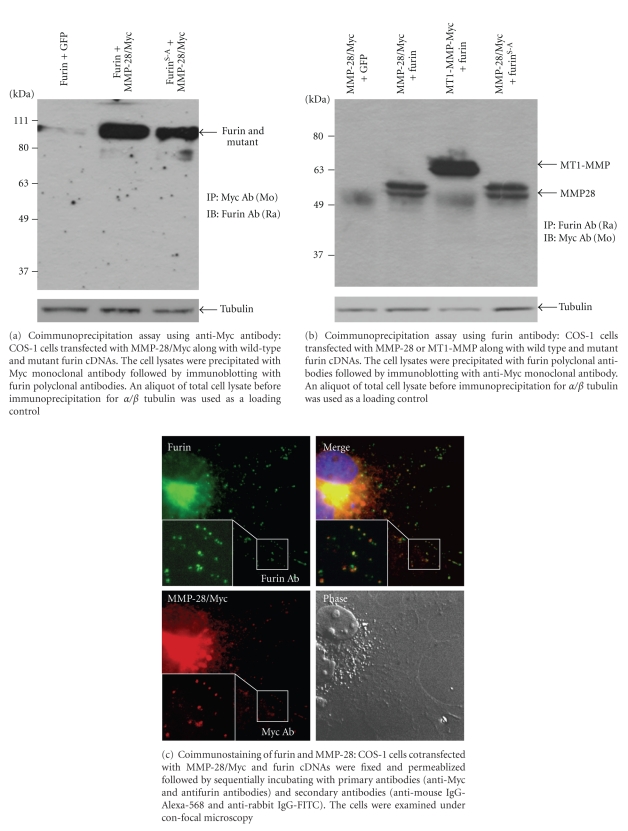
Complex formation between MMP-28 and furin as examined by coimmunoprecipitation and coimmunostaining.

Since furin plays a key nonproteolytic role in MMP-28 secretion, we pursued the possibility that furin may serve as an intermolecular chaperone for MMP-28 trafficking. To this end, COS-1 cells were cotransfected with MMP-28/Myc cDNA along with furin cDNA or GFP cDNA. Cell lysates were collected and immunoprecipitation of MMP-28/Myc complexes was performed using mouse monoclonal anti-Myc antibodies. As shown in Figure 4(a), immunoblotting employing a rabbit antibody to furin revealed the presence of a furin-MMP-28 complex. This interaction was also evident in cells transfected with MMP-28 and constitutively inactive furin^S-A^, suggesting that the interaction between MMP28-Myc and furin is independent of the activation status of furin. In the reciprocal coimmunoprecipitation experiment, MMP28-Myc could also be detected in the furin-immunoprecipitated complex (Figure 4(b)). As a positive control for the co-immunoprecipitation experiment, we confirmed that myc-tagged MT1-MMP forms complexes with furin (Figure 4(b)).

It has been demonstrated that furin is synthesized in the endoplasmic reticulum (ER) and then becomes localized to the TGN and cell surface [40, 41]. Based on our biochemical data demonstrating complex formation between MMP-28 and furin, we employed a double indirect immunofluorescence staining approach followed by confocal microscopy examination to determine the intracellular localization of these proteins (Figure 4(c)). By analyzing staining patterns of MMP-28/Myc and furin in cytoplasmic vesicles from five individual cells and 120 stained vesicles in each cell, an average of 70% MMP-28 was found to colocalize with furin in the same vesicles in transfected COS-1 cells. Taken together, these data suggest that furin interacts with MMP-28 in the secretory vesicles.

### 3.4. Requirement of a Minimal Motif within the Propeptide Domain of MMP-28 for Complex Formation with Furin

**Figure 5:**
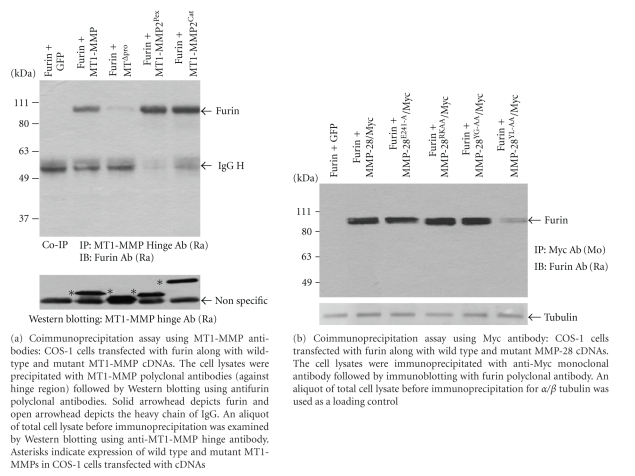
Requirement of the propeptide domain of MT1-MMP for complex formation with furin and the YL sequence of MMP-28 for complex formation and secretion. The YL motif within the propeptide of MMP-28 is necessary.

Furin has been reported to interact with the propeptide domain of MMPs containing RXXR motif between the propeptide domain and the catalytic domain for MMP activations [7, 23]. To determine if furin also interacts with other domains for MMP trafficking, we piloted the experiment by employing our previously generated domain swapping and deletion constructs of MT1-MMP cDNA [33, 42] transfected into COS-1 cells. Coimmunoprecipitation of cell lysates employing an antibody to MT1-MMP, followed by immunoblotting using an antibody to furin, confirmed complex formation between MT1-MMP and furin (Figure 5(a)). Whereas substitution of the catalytic domain (MT1-MMP2^Cat^) or hemopexin domain (MT1-MMP^Pex^) of MT1-MMP with the corresponding regions of MMP-2 cDNA did not alter the complex formation, deletion of the propeptide domain of MT1-MMP (MT^Δpro^) resulted in abrogation of complex formation between furin and MT1-MMP. The failure of interaction between MT1Δpro with furin was not due to lack of MTΔpro expression, as evidenced by a Western blotting using anti-MT1-MMP antibody from an aliquot of the total cell lysate (Figure 5(a), bottom panel). These data are consistent with a requirement for the propeptide in complex formation with furin. 

In order to explore which specific domain of MMP-28 is required for interaction with furin, we compared the amino acid sequences of the propeptide domains of MT1-MMP and MMP-28. This analysis reveals 42% sequence identity between MT1-MMP and MMP-28 (Figure 1). The regions with the highest identity are located in the novel Y^43^GYL^46^ motif, near the C-terminal region containing the PRCGVPD cysteine switch region common to all MMPs, and the C-terminal end containing the RKKR furin consensus sequence. Based on analogy with MT1-MMP, we focused on the propeptide domain and demonstrated that deletion of the entire propeptide domain of MMP-28 resulted in loss of ability of MMP-28 to coimmunoprecipitate with furin (data not shown). To dissect the role of the YGYL motif of MMP-28 in its interaction with furin, cDNAs encoding wild-type and mutant MMP-28 at the YGYL motif were cotransfected with furin followed by coimmunopre cipitation. In the subsequent Western blot depicted in Figure 5(b), the tyrosine-lycine (Y^45^L^46^) sequence in the propeptide of MMP-28 was shown to be critical for complex formation between MMP-28 and furin. Deletion of YGYL in the propeptide domain of MMP-28 also resulted in failure to coimmunoprecipitate with furin (data not shown). Interestingly, mutation of the furin consensus sequence (RKKR) was found to have no effect on furin complex formation. In agreement with the coimmunoprecipitation data, activation of MMP-28 is not a prerequisite for interaction with furin because substitution of the critical catalytic glutamic acid residue with alanine (E-A) [29] of MMP-28 did not interfere with complex formation between mutant MMP-28 and furin. These data highlight the critical role of the YL motif in the propeptide domain of MMP-28 in binding to furin and facilitating MMP-28 secretion.

### 3.5. Furin Enhances MMP-28-Induced Cell Migration

**Figure 6:**
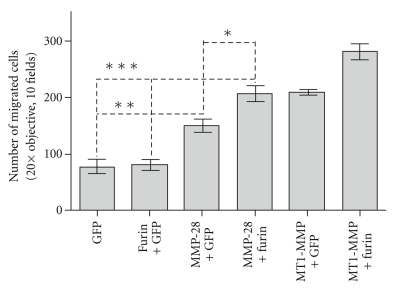
Increased MMP-28 secretion is accompanied with cell migration. The data show that furin enhances cell migration in MMP-28 transfected COS-1 cells. This enhancement is accompanied by elevated MMP-28 trafficking in the secretory pathway. COS-1 cells transfected with cDNAs as indicated were examined by transwell migration assay. Each sample was examined as triplicates and repeated three times. Each bar represents the mean ± SE. (*P = .022;**P = .0058; and ***P = .0005).

We have previously demonstrated that activation of proMMP-28, as well as proMMP-9 and proMT1-MMP, is not a prerequisite for enhanced cell migration through uncoated membranes with 8 *μ*m pores [34]. To assess the functional effect of furin on MMP-28-induced cell migration, COS-1 cells were cotransfected with furin along with MMP-28 cDNA or GFP cDNA control followed by a transwell chamber migration assay. In agreement with our previous observation [34], expression of MMP-28 in COS-1 cells statistically enhanced cell migration as compared to GFP control (*P* = .0058) (Figure 6). MMP-28-induced cell migration was further enhanced by coexpression with furin as compared to furin plus GFP cDNA transfected cells (*P* = .0005). Furin had a similar effect on enhancing the migration of MT1-MMP cDNA transfected COS-1 cells (Figure 6). These results support the concept that furin serves as an intermolecular chaperone to enhanced secretion of MMP-28 and to facilitate cell migration independent of proteolytic activity.

## 4. Discussion

MMP-28, the most recently identified MMP, has been implicated in important biological and pathological processes including neuronal development [26], tissue repair [24], bacterial infection [43], and cancer [29]. In this report, we present a unique mechanism for MMP-28 secretion in which an interaction between the propeptide domain of MMP-28 and furin is a prerequisite for MMP-28 secretion. Contrary to the classical role of furin in cleaving the propeptide of MMPs within a furin consensus sequence, furin-induced cleavage of the propeptide of MMP-28 was not observed. Alanine substituted mutations within the propeptide of MMP-28 demonstrated that furin serves as an intermolecular chaperone for MMP-28 secretion through direct interaction with the Tyr^45^-Leu^46^ motif in the propeptide domain of MMP-28. 

In our initial studies of the release of MMP-28 from transfected COS-1 cells, we encountered unexpected difficulties in detection of MMP-28 in conditioned media. Although MMP-28 was readily detected in the cell lysate, ectopic expression of MMP-28 in COS-1 cells resulted in minimal release of MMP-28 into the conditioned medium. However, coexpression of furin with MMP-28 cDNA in COS-1 cells resulted in markedly enhanced secretion of proMMP-28 (Figure 2(a)). The increase of MMP-28 secretion by furin was not accompanied by upregulation of MMP-28 gene expression (Figure 2(b)). These data indicates that furin enhances trafficking/secretion of MMP-28. 

The YGYL motif is highly conserved in membrane type MMPs and has been found to be essential for MT1-MMP plasma membrane localization [31]. Among secretory MMPs, only MMP-19 and -28 possess this motif in the propeptide domain. The YGYL motif in MMP-28 is conserved among the human, mouse, rat, and *Xenopus laevis* protein [9]. Using mutagenesis analysis, we demonstrated that YL of the YGYL motif is critical for coimmunoprecipitation with furin and for secretion of MMP-28 secretion (Figures 2(d) and 5). 

Cell secretion of MMP-28 has been reported to be variable under different conditions [44]. MMP-28 has been reported to be activated through a furin cleavage mechanism, resulting in a change in molecular mass from ~58 kDa to ~48 kDa [39]. This is consistent with the propeptide domain content of 99 amino acids [9] and a deduced molecular mass of 11.9 kDa. In contrast, we demonstrated that coexpression of furin with proMMP-28 cDNA in COS-1 cells did not lead to the activation of MMP-28. Although we identified a doublet of MMP-28 in Western blot analysis of transfected cell lysates and conditioned media, the difference in mass of the doublet was ~5 kDa (Figure 2). Employing Endo H and PNGase F, we demonstrated that the upper band represents a glycosylated form of MMP-28 (Figure 3). Murine MMP-28 has also been reported to be N-glycosylated [39]. 

Secretion of MMP-28 into conditioned media as a furin-cleaved protein (Mr-48 kDa) has been reported with human HT1080 fibrosarcoma, HeLa cervical cancer cells and A549 lung adenocarcinoma cells transfected with mouse MMP-28 cDNA [29, 39, 44]. In contrast, Western blot analysis of conditioned media isolated from transiently transfected CHO cells identified latent MMP-28 protein bands of 58, 57, and 52 kDa; activated MMP-28 was not observed [39]. The discrepancy between absence of activated MMP-28 in transfected nonmalignant COS-1 cells (Figure 2) and CHO cells as compared to activated MMP-28 in transfected cancer cells cannot be readily explained. Theoretically, preprotein convertase furin is responsible for activation of MMPs containing furin-consensus sequence [6]. Growing evidence also indicate that cleavage of MMP, that is MT1-MMP, at furin consensus sequence is not a prerequisite for the activation of MT1-MMP [30, 31, 35, 45, 46]. Employing a furin inhibitor, Dec-CMK, in MT1-MMP expressing VSMCs, Kappert et al. [47] demonstrated that MT1-MMP enzymatic activity was not affected by furin inhibitor, suggesting furin is not required for the MT1-MMP/MMP-2 proteolytic activation cascade in primary VSMCs. A furin-independent pathway of MT1-MMP activation has been proposed [48, 49]. These differences reflect the complication of molecular and cellular biology and detailed mechanism underlying these difference remains to be understood. 

In this report, we demonstrated that furin forms a complex with both MMP-28 and MT1-MMP, involving their propeptide domains (Figure 5). This interaction appears to facilitate MT1-MMP plasma membrane trafficking and MMP-28 secretion [30, 31, 35, 36]. Although MMP-28 contains a paired basic amino acid (RKKR) furin cleavage site between the propeptide and catalytic domains, we found that mutation at this site in MMP-28 did not impair furin-enhanced MMP-28 secretion. Furthermore, constitutively inactive furin possessed unabated ability to enhance MMP-28 secretion. Confocal microscopy showed that the co-localization of furin and MMP-28 was detected intracellularly, primarily in the perinuclear region. Although this co-localization staining is not in itself conclusive of an interaction between MMP-28 and furin, our biochemical data together with the immunocytochemical staining data demonstrate that MMP-28 indeed interacts with furin in the secretory pathway. Our data are in agreement with previous reports showing that furin-induced cleavage at furin consensus sequences of proproteins [IFN-*γ* [50], zona pellucida glycoprotein ZP3[50], proneurotrophin-3 [51]] is not necessary for proprotein trafficking/secretion. 

MMP-28 has been reported to both enhance [34] and diminish [44] cell migration. Illman et al. reported that enzymatic activity of MMP-28 in human A549 lung carcinoma is not a prerequisite for enhanced cell migration [29]. In agreement with these observations, we demonstrated that transfection of MMP-28 cDNA into COS-1 cells resulted in enhanced migration; furin co-transfection further facilitated cell migration. In contrast, Manicone et al. [43] used a MMP-28-null mouse pneumonia model to demonstrate that MMP-28 restrains the migration of macrophages; unidentified factors in serum were implicated [52]. 

In conclusion, we observed a novel role for furin serving as a chaperone for MMP-28 secretion. This enhanced secretion is based on interaction of furin with the YL motif in the propeptide domain of MMP-28. These findings add to the list of novel functions of furin and distinct characteristics of the YGYL domain of selected MMPs. 

## Abbreviations

MMPs: matrix metalloproteinases
Endo H: Endoglycosidase H
PNGase F: Peptide N*-*Glycosidase F
IFN-*γ*: Interferon*-*gamma
TGN: *trans *Golgi network
HPRT: hypoxanthine ribosyltransferase
G6PDH: Glucose-6-phosphate dehydrogenase.
